# Re-examining the factor structure of the Insomnia Severity Index (ISI) and defining the meaningful within-individual change (MWIC) for subjects with insomnia disorder in two phase III clinical trials of the efficacy of lemborexant

**DOI:** 10.1186/s41687-024-00744-6

**Published:** 2024-06-29

**Authors:** William R. Lenderking, Yulia Savva, Mark J. Atkinson, Renee Campbell, Isabelle Chabot, Margaret Moline, Genevieve Meier, Charles M. Morin

**Affiliations:** 1 Patient-Centered Research, Evidera, Bethesda, MD USA; 2 Formerly at Evidera, Bethesda, MD USA; 3 Eisai, Nutley, NJ USA; 4 Faculty of Pharmacy, University of Montreal, Montreal, QC Canada; 5 School of Psychology and Brain Research Center, Laval University, Quebec City, Canada

**Keywords:** Clinically meaningful change, Confirmatory factor analysis, Insomnia, Insomnia Severity Index, Lemborexant, Meaningful within-individual change, Validity

## Abstract

**Background:**

The Insomnia Severity Index (ISI) is a widely used measure of insomnia severity. Various ISI research findings suggest different factor solutions and meaningful within-individual change (MWIC) to detect treatment response in patients with insomnia. This study examined an ISI factor solution and psychometric indices to define MWIC in a robust patient sample from clinical trial settings.

**Methods:**

We endeavored to improve upon previous validation of ISI by examining structural components of confirmatory factor analysis (CFA) models using two large, placebo-controlled clinical trials of lemborexant for insomnia. Using the best-fitting two-factor solution, we evaluated anchor-based, distribution-based and receiver operating characteristic (ROC) curve methods to derive an estimate of the MWIC.

**Results:**

The model structure for the 7-item scale proposed in other research did not fit the observed data from our two lemborexant clinical trials (*N* = 1956) as well as a two-factor solution based on 6 items did. Using triangulation of anchor-based, distribution-based, and ROC methods, we determined that a 5-point reduction using 6 items best represented a clinically meaningful improvement in individuals with insomnia in our patient sample.

**Conclusions:**

A 6-item two-factor scale had better psychometric properties than the 7-item scale in this patient sample. On the 6-item scale, a reduction of 5 points in the ISI total score represented the MWIC. Generalizability of the proposed MWIC may be limited to patient populations with similar demographic and clinical characteristics.

**Supplementary Information:**

The online version contains supplementary material available at 10.1186/s41687-024-00744-6.

## Background

Insomnia is a widespread problem, with studies estimating a global prevalence of chronic insomnia between 10% and 15% of the population, with an additional 25–35% suffering from transient insomnia [1]. It is characterized by persistent difficulties with initiating sleep at bedtime, frequent or prolonged awakenings, or early-morning awakenings with inability to return to sleep, despite adequate opportunities to sleep, or some combination of these problems [2]. Such sleep disruptions result in clinically significant impairment of daytime function, including fatigue, decreased energy, mood disturbances, and reduced cognitive function.

The Insomnia Severity Index (ISI) is a well-known and widely used patient-reported outcome (PRO) measure used to assess insomnia symptoms and daily functioning in clinical trials. The reliability and validity of the ISI have previously been established, however, the factorial structure remains a topic of active interest [3–7]. For example, one study of adult insomnia patients in Taiwan, China, and Canada found the ISI to have three factors, while another study in Chinese adolescent patients found a structure of two factors [4, 5]. Other findings suggest that the ISI items may not all similarly contribute to overall insomnia severity, at least in some selected patient populations [6].

In addition to variability in the proposed factor structures and resulting ambiguity about scoring of domains, there is no consensus on the threshold(s) for clinically meaningful change. The few studies that have attempted to define a threshold for the clinical meaningfulness of changes on the 7-item ISI report estimates ranging between 6-point and 8.4-point reductions [6, 7]. Further research on the interpretability of the ISI scores is also important for clinical research on insomnia treatment.

The primary objectives of the current analysis were to evaluate the ISI factor structure using confirmatory factor analysis (CFA), and then to evaluate the psychometric properties of the ISI based on the factor structure that best fitted our patient sample. A secondary objective of this analysis was to evaluate thresholds for the clinically meaningful within-individual change (MWIC) of the scores derived from the revised ISI.

## Methods

### Study design

We used data from two Eisai pivotal trials of lemborexant (LEM) for insomnia disorder. SUNRISE-1 (NCT02783729; E2006-G000-304) was a 1-month, double-blind, randomized, placebo (PBO)- and active-controlled, parallel-group study [8]. SUNRISE-2 (NCT02952820; E2006-G000-303) was a 12month (6-month PBO-controlled, followed by 6-month active treatment only) double-blind study [9]. In both clinical trials, the ISI was completed at baseline, at the end of Month 1, and for SUNRISE-2, at Months 3, 6, and 12. The baseline ISI values were derived just prior to the mid-run-in polysomnogram in SUNRISE-1 and from the scores at the end of the PBO run-in for subjects in SUNRISE-2.

### Study population

Participants included in this analysis were 1006 participants (ages: women ≥ 55 years and men ≥ 65 years) in SUNRISE-1 and 950 participants (age ≥ 18 years) in SUNRISE-2. Participants met Diagnostic and Statistical Manual of Mental Disorders, 5th edition criteria for insomnia disorder. Individuals diagnosed with comorbid sleep disorders including sleep apnea, periodic limb movement disorder, restless leg syndrome, circadian rhythm sleep disorder or narcolepsy, and individuals with a history of complex sleep -related behavior, were not eligible for the trials. However, subjects with stable medical or psychiatric conditions that would not interfere with participating in the study were eligible to participate.

### Measures

The ISI is a patient-reported 7-item questionnaire assessing insomnia symptoms and its impact on daytime functioning. Patients are asked to think about their *current (i.e. last two weeks)* insomnia symptoms and impacts. The items are focused on difficulties related to sleep onset and maintenance, problems related to early-morning awakening, satisfaction with sleep pattern, noticeability of sleep problems by others, degree of distress or concern caused by sleep difficulties, and interference of sleep difficulties on daily functioning [6]. The items can be rated from 0 to 4 (0 = no problem; 4 = very severe problem), with a total score ranging from 0 to 28. The total score can be interpreted as follows: absence of insomnia (0–7), subthreshold insomnia (8–14), moderate insomnia (15–21), and severe insomnia (22–28). Previous research has identified at least two clinically important thresholds for the ISI total score: Morin et al. proposed a change of −8.4 points as a moderate improvement, while Yang et al. proposed a change of −6 points as a minimally clinically important difference [6, 7].

Patient Global Impression–Insomnia (PGI-I) provides a self-reported questionnaire assessing the patients’ perception of a medication’s effects on sleep after treatment compared with their sleep prior to treatment initiation [10]. Hence, the three PGI-I items are related to the benefit of study medication as perceived by patients: (i) Helped/Worsened Sleep, (ii) Increased/Decreased Time to Fall Asleep, and (iii) Increased/Decreased Total Sleep Time (TST). Each of these items are rated on a 3-point categorical scale for medication effect: positive, neutral, and negative. PGI-I scores at Month 1 were utilized for the analyses presented here.

### Statistical analysis

Demographic and clinical characteristics of the patient sample at baseline were calculated using mean, standard deviation (SD), and range for continuous variables, and percentages and frequencies for categorical variables.

We examined the distribution of the responses on the 7 items of the ISI as well as the mean and median for the ISI total score, Insomnia Symptoms, and Daytime Functioning scores at baseline. Additionally, the change in ISI total score from baseline to Month 1 was assessed in each treatment group.

A CFA is used in psychometric research to test if data fit a hypothesized model [11, 12]. We hypothesized that a two-factor solution would best fit the ISI model based on an evaluation of the content of the scale: the first three items measure different insomnia patterns (difficulty falling asleep, remaining asleep, and early-morning awakening), while the remaining four measure daytime functioning. We conducted a CFA to assess the best-fitting factor structure of the ISI and then used the scoring algorithm that was defined based on the results of the CFA. To evaluate the factor structure, the comparative fit index (CFI, minimum threshold of 0.9) and root mean square error of approximation (RMSEA, maximum acceptable threshold of 0.09) were examined. In our approach, we also used modification indices (MIs) to demonstrate an improvement in model fit when the suggested modifications to the model specifications were introduced. The MI indicated how much the model could be improved if a particular path were added to the model or a constraint removed; a value of > 3.84 is considered to represent a statistically significant improvement [11]. In this approach, by removing an item with residual correlations, we increased the model precision by reducing measurement error. This statistical approach was largely driven by the US Food and Drug Administration (FDA) guidance for PRO, which suggests that a PRO instrument should be “fit for purpose” for the target population [13].

To define a threshold for a MWIC, a multistep triangulation approach was used. Anchor-based methods were utilized to determine the possible range, then receiver operating characteristic (ROC) methods were used to refine the range to a single value. Distribution-based methods were evaluated as supplementary for the MWIC. The anchor-based method was based on three items from the PGI-I. The anchor-based method is a preferential method according to the FDA [13] given that it directly considers the patient’s voice to determine within-individual change. Within-individual change is the average amount of change experienced by patients within the same group or treatment arm.

The distribution-based methods included half the SD at baseline and the standard error of measurement (SEM). Although these are group-based methods, they can still be used as supportive in establishing measures of change within individuals.

The ROC method compared the predictive power for different ISI cutoffs defined using the three PGI-I items (Helped/Worsened Sleep, Increased/Decreased Time to Fall Asleep, and Increased/Decreased Total Sleep Time). For each ROC analysis, the dependent variable “Responder” was defined as “positive medication effect” on the PGI-I. The optimal cutoffs were selected using the data on sensitivity, specificity, and the Youden index. The Youden index is often used as a criterion to select optimal cutoff values across varying levels of sensitivity and specificity. Technically, it gives equal weights to false-positive and false-negative values and thus provides information on the proportion of the total misclassified for a specific cutoff, which can then be compared across different cutoffs. This index ranges from 0 to 1, and the highest value serves as an indicator of an optimal test performance. The probability density function (PDF) by treatment arms has also been used as supporting evidence to establish the threshold for clinical meaningfulness (Fig. 2).

The main objectives of the study were to re-examine the factor structure of the ISI instrument and to define the MWIC for the targeted population—individuals with insomnia disorder from the two clinical trials, SUNRISE-1 and SUNRISE-2.

## Results

### Patient demographics and clinical characteristics

The mean age of the pooled study population was 59.3 (SD 11.8), and the majority were female (77.6%) and White (71.9%) (Table 1). In the two trials, the mean ages and range were as follows: SUNRISE-1: mean age: 63.93 (SD 6.81) and range: 55–88; SUNRISE-2: mean age: 54.49 (SD 13.8) and range: 18–88. At baseline, the mean ISI score was 18.8 (SD 3.7) in the pooled trials, indicating that, on average, participants of both studies had moderately severe insomnia, based on their self-perception (Table 2). In both SUNRISE-1 and SUNRISE-2, patients’ responses to individual item scores within Factor 1 indicated that the majority of patients also had moderate to very severe insomnia according to the three patterns of sleep disturbance: sleep-onset problems (90.6%); sleep maintenance difficulties (98%); and early-morning awakening problems (88.2%) (Supplementry Table 1). The impact of insomnia on patients was large at baseline according to the Factor 2 items: 94.0–97.7% of subjects across the two studies reported to be dissatisfied or very dissatisfied with current sleep pattern (item 4); 29.5–37.8% thought their sleep problem was much or very much noticeable by others (item 5); 65.9–73.0% had much or very much distress or concern caused by sleep difficulties (item 6); and 45.0–56.9% had much or very much interference of sleep difficulties with daily functioning (item 7) (Supplementry Table 1).

**Table 1:** Demographic characteristics and sleep metrics at baseline

Characteristic	SUNRISE-1 (*N* = 1006)	SUNRISE-2 (*N* = 950)	Pooled trials (*N* = 1956)
Age			
Mean (SD)	63.93 (6.81)	54.49 (13.80)	59.34 (11.77)
Median (Q1–Q3)	63.0 (58.00–68.00)	55.0 (44.00–66.00)	60.0 (55.00–67.00)
Range (min–max)	(55.00–88.00)	(18.00–88.00)	(18.00–88.00)
Sex			
Male	137 (13.6%)	302 (31.8%)	439 (22.4%)
Female	869 (86.4%)	648 (68.2%)	1517 (77.6%)
Race			
White	727 (72.3%)	680 (71.6%)	1407 (71.9%)
Black or African American	256 (25.4%)	76 (8.0%)	332 (17.0%)
Asian	14 (1.4%)	178 (18.7%)	192 (9.8%)
American Indian or Alaska Native	0 (0.0%)	3 (0.3%)	3 (0.2%)
Native Hawaiian or other Pacific Islander	2 (0.2%)	1 (0.1%)	3 (0.2%)
Other^1^	7 (0.7%)	12 (1.3%)	19 (1.0%)
Sleep diary variables			
Subjective sleep-onset latency (minutes)			
N	–	943	943
Mean (SD)	–	64.04 (45.93)	64.04 (45.93)
Median (Q1–Q3)	–	55.4 (33.57–79.29)	55.4 (33.57–79.29)
Range (min–max)	–	(3.57–445.7)	(3.57–445.7)
Subjective total sleep time (minutes)			
N	–	909	909
Mean (SD)	–	308.7 (91.10)	308.7 (91.10)
Median (Q1–Q3)	–	320.6 (252.1–374.8)	320.6 (252.1–374.8)
Range (min–max)	–	(0.00–531.2)	(0.00–531.2)
Subjective wake-after-sleep onset (minutes)			
N	–	939	939
Mean (SD)	–	133.9 (83.41)	133.9 (83.41)
Median (Q1–Q3)	–	117.6 (71.00–182.9)	117.6 (71.00–182.9)
Range (min–max)	–	(0.00–460.0)	(0.00–460.0)
Polysomnography			
Average of latency to persistent sleep (minutes)			
N	1005	–	1005
Mean (SD)	44.50 (35.46)	–	44.50 (35.46)
Median (Q1–Q3)	34.3 (19.00–61.25)	–	34.3 (19.00–61.25)
Range (min–max)	(0.50–267.0)	–	(0.50–267.0)
Average of total sleep time (minutes)			
N	1005	–	1005
Mean (SD)	327.5 (52.42)	–	327.5 (52.42)
Median (Q1–Q3)	335.8 (299.8–367.5)	–	335.8 (299.8–367.5)
Range (min–max)	(96.50–416.5)	–	(96.50–416.5)
Average of wake-after-sleep onset (minutes)			
N	1005	–	1005
Mean (SD)	113.7 (39.09)	–	113.7 (39.09)
Median (Q1–Q3)	106.3 (83.75–134.8)	–	106.3 (83.75–134.8)
Range (min–max)	(37.25–286.8)	–	(37.25–286.8)

*min* minimum, *max* maximum, *Q* quartile, *SD* standard deviation

^1^“Other” race included: African American/American Indian (*n* = 1); American Indian, Black, White, Hispanic (*n* = 1); Biracial–White and African American (*n* = 1); Black and White (*n* = 1); Black and American Indian (*n* = 1); Caucasian and African American (*n* = 1); European (*n* = 1); Hispanic (*n* = 1); Latin American Heritage (*n* = 1); Mixed–American Indian and White (*n* = 1); Refused to Report (*n* = 1); Unknown (*n* = 1); White/American Indian or Alaska Native (*n* = 1); White Native American and African American (*n* = 1); White and Native Hawaiian/Pacific Islander (*n* = 1); Black, White, America Indian (*n* = 1); Mixed (*n* = 1); Multiracial (*n* = 1); Puerto Rican (*n* = 1)

**Table 2:** Description of baseline ISI total and daytime functioning scores in SUNRISE-1 and SUNRISE-2 (*N* = 1956)

ISI scores	SUNRISE-1 (*N* = 1006)	SUNRISE-2 (*N* = 950)	Pooled trials (*N* = 1956)
Insomnia symptoms			
Mean (SD)	8.00 (1.75)	8.07 (1.78)	8.04 (1.77)
Median	8.0	8.0	8.0
Range (min–max)	1.00–12.00	2.00–12.00	1.00–12.00
Q1–Q3	7.00–9.00	7.00–9.00	7.00–9.00
Daytime functioning			
Mean (SD)	10.39 (2.81)	11.15 (2.10)	10.76 (2.52)
Median	11.0	11.0	11.0
Range (min–max)	0.00–16.00	1.00–16.00	0.00–16.00
Q1–Q3	8.00–12.00	10.00–12.00	9.00–12.00
Total score			
Mean (SD)	18.40 (3.97)	19.22 (3.26)	18.80 (3.66)
Median	18.0	19.0	19.0
Range (min–max)	1.00–28.00	4.00–28.00	1.00–28.00
Q1–Q3	16.00–21.00	17.00–21.00	16.00–21.00

Data are based on the original 7-item ISI total score

*ISI* Insomnia Severity Index, *Q* quartile, *SD* standard deviation

### Change from baseline at Day 31/Month 1

After 1 month of treatment, the active treatment groups showed a higher proportion of patients dropping two or three categories of insomnia severity compared with PBO (a shift down by one category of severity is defined as a clinically significant benefit from treatment) (Table 3). For the pooled trial, at Day 31/Month 1, the percentage of subjects shifted to the moderate category (ISI total score 15–21) were 45.4% for PBO, 32.9% for lemborexant 5 mg (LEM5), 23.0% for lemborexant 10 mg (LEM10), and 31.7% for zolpidem tartrate (ZOL). A smaller but still considerable number of subjects jumped over two categories and shifted to “subthreshold insomnia” (ISI total score 8–14): 23.1% for PBO, 27.3% for LEM5, 31.1% for LEM10, and 31.7% for ZOL. A few subjects jumped over three categories and shifted to the “No clinically significant insomnia” (ISI total score 0–7): 12.0% for PBO, 24.5% for LEM5, 32.8% for LEM10, and 31.7% for ZOL.

**Table 3:** Change in ISI total score from baseline to Month 1/Day 31

	**Baseline ISI total score (*N*** **=** **1956)**															
	0–7				8–14				15–21				22–28			
	PBO *n* = 4	LEM5 *n* = 1	LEM10 *n* = 2	ZOL *n* = 0	PBO *n* = 42	LEM5 *n* = 46	LEM10 *n* = 55	ZOL *n* = 46	PBO *n* = 365	LEM5 *n* = 389	LEM10 *n* = 397	ZOL *n* = 151	PBO *n* = 117	LEM5 *n* = 146	LEM10 *n* = 129	ZOL *n* = 66
Month 1	*n* = 4	*n* = 1	*n* = 2	*n* = 0	*n* = 41	*n* = 44	*n* = 46	*n* = 41	*n* = 342	*n* = 370	*n* = 369	*n* = 143	*n* = 108	*n* = 143	*n* = 122	*n* = 60
0–7 (*n* = 413)	4 (100.0%)	1 (100.0%)	2 (100.0%)	0 (0.0%)	8 (19.5%)	14 (31.8%)	18 (39.1%)	16 (39.0%)	41 (12.0%)	89 (24.1%)	80 (21.7%)	33 (23.1%)	13 (12.0%)	35 (24.5%)	40 (32.8%)	19 (31.7%)
8–14 (*n* = 792)	0 (0.0%)	0 (0.0%)	0 (0.0%)	0 (0.0%)	27 (65.9%)	27 (61.4%)	26 (56.5%)	21 (51.2%)	159 (46.5%)	174 (47.0%)	163 (44.2%)	74 (51.7%)	25 (23.1%)	39 (27.3%)	38 (31.1%)	19 (31.7%)
15–21 (*n* = 543)	0 (0.0%)	0 (0.0%)	0 (0.0%)	0 (0.0%)	5 (12.2%)	3 (6.8%)	2 (4.3%)	4 (9.8%)	135 (39.5%)	101 (27.3%)	118 (32.0%)	32 (22.4%)	49 (45.4%)	47 (32.9%)	28 (23.0%)	19 (31.7%)
22–28 (*n* = 88)	0 (0.0%)	0 (0.0%)	0 (0.0%)	0 (0.0%)	1 (2.4%)	0 (0.0%)	0 (0.0%)	0 (0.0%)	7 (2.0%)	6 (1.6%)	8 (2.2%)	4 (2.8%)	21 (19.4%)	22 (15.4%)	16 (13.1%)	3 (5.0%)

FAS in pooled trials (*N* = 1956)

Percentages are column percentages. Data are based on the original 7-item ISI total score. Missing values are not included in the denominator for percentages

In SUNRISE-1, the baseline ISI total score could have been 13 + in SUNRISE-1 but 15 + in SUNRISE-2. For subjects with scores below entry criteria on the ISI, a very small number of subjects were enrolled who otherwise met study entry criteria but did not meet ISI entry criteria (< 0.04%)

*FAS* full analysis set, *ISI* Insomnia Severity Index, *LEM5* lemborexant 5 mg, *LEM10* lemborexant 10 mg, *PBO* placebo, *ZOL* zolpidem tartrate

### Confirmatory factor analysis

**Fig. 1:**
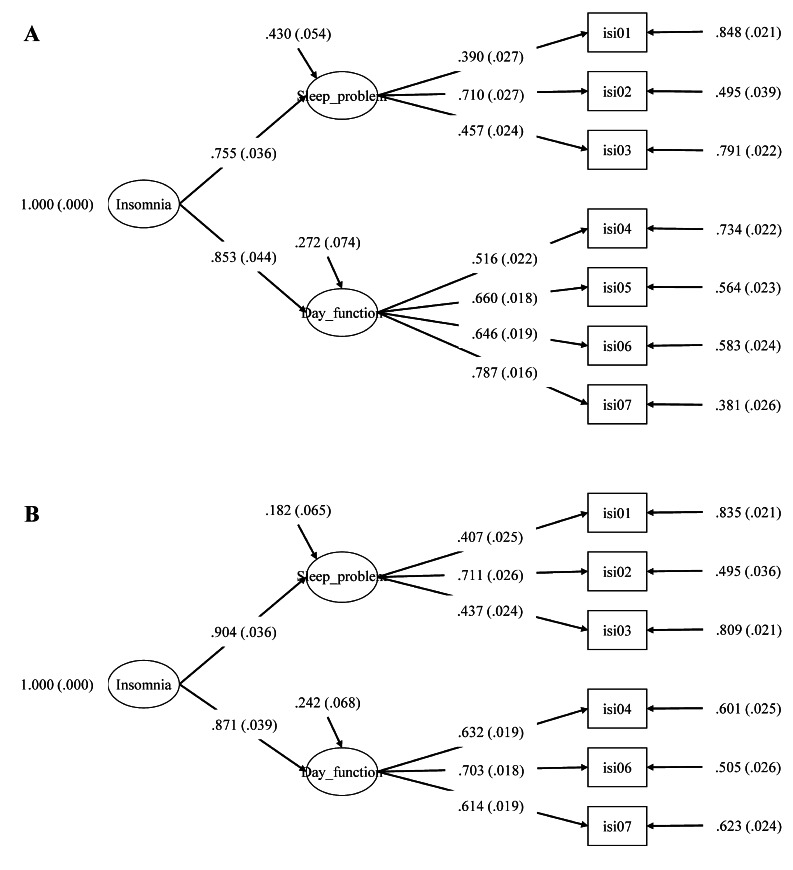
Two-factor confirmatory factor analysis models (*N* = 1956) **A** with 7 items; **B** with item 5 removed. The figures show the latent constructs of sleep problems and daytime functioning (Factors 1 and 2), and the item factor loadings on each item of the ISI

A preliminary CFA was conducted on the pooled trial dataset using the prespecified seven ISI items in our two-factor measurement model (Factor 1, items 1–3; Factor 2, items 4–7) (Table 4 and Fig. 1A). Figure 1A shows the hypothesized two-factor structure along with the item loadings on each factor. For this model, goodness of fit indices of the 7-item model indicated weak structure with a CFI of 0.862 and excessive model error with an RMSEA of 0.126 (90% confidence interval [CI]: 0.116–0.137; Table 4). Examination of the highest MIs for this model revealed the presence of very highly correlated residuals between ISI items 5 (noticeability of sleep problem to others) and 7 (extent to which current sleep problem interferes with daily functioning) (MI = 348.8, which was much larger than the cut-off for significance of 3.84) (Table 5).

**Table 4:** Confirmatory factor analysis of the ISI with and without item 5

Item	CFA factor loadings for CFA model (7 items)^1^		CFA factor loadings for CFA model (6 items)	
	Factor 1^2^	Factor 2^3^	Factor 1^2^	Factor 2^3^
1. Difficulty falling asleep	0.390	–	0.407	–
2. Difficulty staying asleep	0.710	–	0.711	–
3. Problems waking up too early	0.457	–	0.437	–
4. How satisfied/dissatisfied are you with your current sleep pattern?	–	0.516	–	0.632
5. How noticeable to others do you think your sleep problem is in terms of impairing the quality of your life?	–	0.660	–	–
6. How worried/distressed are you about your current sleep problem?	–	0.646	–	0.703
7. To what extent do you consider your sleep problem to interfere with your daily functioning (e.g. daytime fatigue, mood, ability to function at work/daily chores, concentration, memory, mood, etc.) currently?	–	0.787	–	0.614
Factor loadings on general factor				
F1 loading	0.755		0.904	
F2 loading	0.853		0.871	
Model fit statistics				
Chi-square (df)	419.89 (13)		95.76 (8)	
*P*-value	0.0000		0.0000	
CFI	0.862		0.956	
RMSEA	0.126		0.075	
90% CI	(0.116–0.137)		(0.062–0.089)	
Test of close fit: *p* ≤ 0.05	0.000		0.001	
WRMR	0.062		0.033	

Standardized factor loadings at baseline using the FAS in the pooled trials (*N* = 1956)

Dropping of ISI item 5 from the base model provided a model that did not require any modifications to obtain reasonable fit statistics and insomnia items with factor loadings over 0.40

^1^Considered the base (original) model

^2^Factor 1 was characterized by items indicative of more insomnia symptoms

^3^Factor 2 was characterized by items indicative of a more impaired daytime functioning

*CFA* confirmatory factor analysis, *CFI* comparative fit index, *CI* confidence interval, *ISI* Insomnia Severity Index, *RMSEA* root mean square error of approximation, *WRMR* weighted root mean square residual

**Table 5:** Confirmatory factor analysis of ISI within-factor item–item modification indices, FAS in pooled trial (*N* = 1956)

	Variable 1	Variable 2	Modification index	EPC	Std YX EPC
Base model + 1 modification	5. How noticeable to others do you think your sleep problem is in term of impairing the quality of your life?	7. To what extent do you consider your sleep problem to interfere with your daily functioning?	348.84	0.376	0.928

*EPC* expected parameter change

*Note* Std YX EPC. stands for standardized expected parameter change indices

As a result of this analysis, it seemed appropriate to drop item 5 (0.660) instead of item 7 (0.787) to improve model fit, given the lower factor loading associated with item 5 (Table 6). It also made sense conceptually, as item 5 is the only item that does not ask for a direct self-assessment compared with the other items; rather, it asks the respondent to rate how they believe others perceive them. This is potentially more prone to inaccuracy than a self-assessment might be, decreases precision of the instrument, and in return “weakens” the outcome summary measure [14].

**Table 6:** Anchor-based definition of meaningful change from baseline to Month 1/Day 31

Change in ISI score	Medication effect, item 1 of PGI-I: Helped/worsened sleep		
	Positive (*n* = 1042)	Neutral (*n* = 422)	Negative (*n* = 372)
Total score			
F-value 384 (*p* < 0.0001)			
n	1042	422	372
Mean (SD)	−8.15 (4.98)	−3.59 (3.37)	−1.67 (2.91)
Median	−8.0	−3.0	−2.0
SE	0.15	0.16	0.15
Q1–Q3	(−12.0 to −5.00)	(−6.00 to −1.00)	(−3.00 to 0.00)
Min to max	(−24.0 to 7.00)	(−19.0 to 5.00)	(−13.0 to 8.00)

Table results based on 6-item ISI using FAS (*N* = 1956)

*FAS* full analysis set, *PGI-I* Patient Global Impression– Insomnia, *Q* quartile, *SD* standard deviation, *SE* standard error of mean

The reduced 6-item model without item 5 demonstrated an acceptable model fit. The CFI improved substantially to 0.956 and exceeded the minimum threshold value of 0.9; the RMSEA was below the maximum acceptable threshold (< 0.09) at 0.075 (90% CI: 0.062–0.089). All loadings were above 0.4 (Table 4 and Fig. 1B). The 6-item model included a two-factor structure, with a factor loading on the overall general factor of 0.904 for the Insomnia Symptoms domain (items 1–3) and 0.871 for the Daytime Functioning domain (items 4, 6, 7).

### Threshold for clinically meaningful change

Based on the findings from the CFA, the results below are provided for the 6-item ISI total score, omitting item 5.

#### Anchor-based methods

The PGI-I, which was utilized for the anchor-based methods, had moderate to strong correlations with the PGI-I items at Day 31/Month 1 (*r* = 0.43 to 0.60; *p* < 0.0001).

The anchor-based method with the PGI-I item “Helped/Worsened Sleep” demonstrated that the mean changes (SD) in ISI total score for medication effect at Day 31/Month 1 for the pooled trials were as follows: positive, −8.15 (4.98); neutral, −3.59 (3.37); and negative, −1.67 (2.91) (Table 6). When used with the PGI-I item “Reduced Time to Fall Asleep,” the anchor-based method demonstrated mean changes in ISI total score of −7.49 (5.10) for positive, −4.54 (4.13) for neutral, and −2.94 (4.04) for negative medication effects at Day 31/Month 1. The estimates for “Increased Total Sleep Time” were −8.15 (5.04) for positive, −3.74 (3.23) for neutral, and −2.40 (3.49) for negative medication effects.

The mean changes in ISI total scores for the neutral and positive medication effects responses suggested that the MWIC for the 6-item total ISI, should be in the range of −4 to −8. This wide range is associated with the fact that the anchors from the PGI-I item have only three levels, which include negative, neutral, and positive medication effects. Given that the positive effect is an average of all “positive medication effect” responses, a positive effect would include both large positive and minimal positive effects, which suggests the MWIC value should be between the neutral and the positive range.

#### Distribution-based methods

Distribution-based methods were applied to provide evidence about measurement variability per FDA recommendations [13]. The SEM for the 6-item total ISI score was determined to have a value of 1.70. Additionally, the 0.5 SD at baseline was 1.55 for the pooled trials (Table 7). The distribution-based methods gave a much more restricted estimate of the MWIC, suggesting an MWIC of between 0 and −1.5.

**Table 7:** Distribution-based methods to identify change thresholds from baseline to Month 1/Day 31

Change in ISI score	Baseline Cronbach’s alpha (*N* = 1956)	SEM (*N* = 1956)	0.5*SD at baseline (*N* = 1956)	ES	Anchor-based change from baseline to Month 1/Day 31 Mean (SD)
Insomnia symptoms	0.47	1.29	0.88	−1.54	−3.78 (2.58)
Daytime functioning	0.68	1.04	0.92	−1.68	−4.37 (2.87)
Total score	0.70	1.70	1.55	−1.87	−8.15 (4.98)

ES was calculated as the change from baseline divided by the SD at baseline for the sample at Month 1. Anchorbased change from baseline to Month 1 was for positive medication effect. SEM and 1.5 SD at baseline were calculated using the FAS *N* = 1956

*ES* effect size, *FAS* full analysis set, *ISI* Insomnia Severity Index, *SD* standard deviation, *SEM* standard error of measurement

#### ROC method

The ROC analysis showed that the cutoff at the ISI total score up to −5 had the highest Youden’s index (0.49), compared with the other cutoffs, to predict the individuals with the positive medication effect on the PGI-I item “Helped/Worsened Sleep” using the data for the pooled trial at Day 31/Month 1 (Table 8). The sensitivity and specificity were 0.76 and 0.74, respectively. An MWIC of −5 falls within the range observed for PGI-I items “Reduced Time to Fall Asleep” and “Increased Total Sleep Time.” Thus, these approaches provided strong evidence for an MWIC of −5 for the ISI total score.

**Table 8:** ROC analysis of the change in 6-item ISI total score predicting PGI-I Item 1 at Month 1

Average change in ISI total score	Sensitivity	Specificity	Positive predictive value	Negative predictive value	Youden index	Phi^1^
≤ −8	0.52	0.92	0.90	0.60	0.45	0.47
≤ −7	0.59	0.87	0.86	0.62	0.47	0.47
≤ −6	0.67	0.81	0.82	0.65	0.48	0.47
≤ −5	0.76	0.74	0.79	0.70	0.49	0.49
≤ −4	0.82	0.64	0.75	0.73	0.46	0.47
≤ −3	0.88	0.51	0.70	0.76	0.39	0.42
≤ −2	0.91	0.37	0.65	0.76	0.28	0.34
≤ −1	0.95	0.27	0.63	0.80	0.22	0.31

^1^Phi correlation examines association between ISI total score and PGI-I

PGI-I Item 1 is “Helped/Worsened Sleep”

Youden’s Index is sensitivity + (specificity −1)

*ISI* Insomnia Severity Index, *PGI-I* Patient Global Impression–Insomnia, *ROC* receiver operating characteristic

#### Evaluating treatment differences in the context of the proposed MWIC

**Fig. 2:**
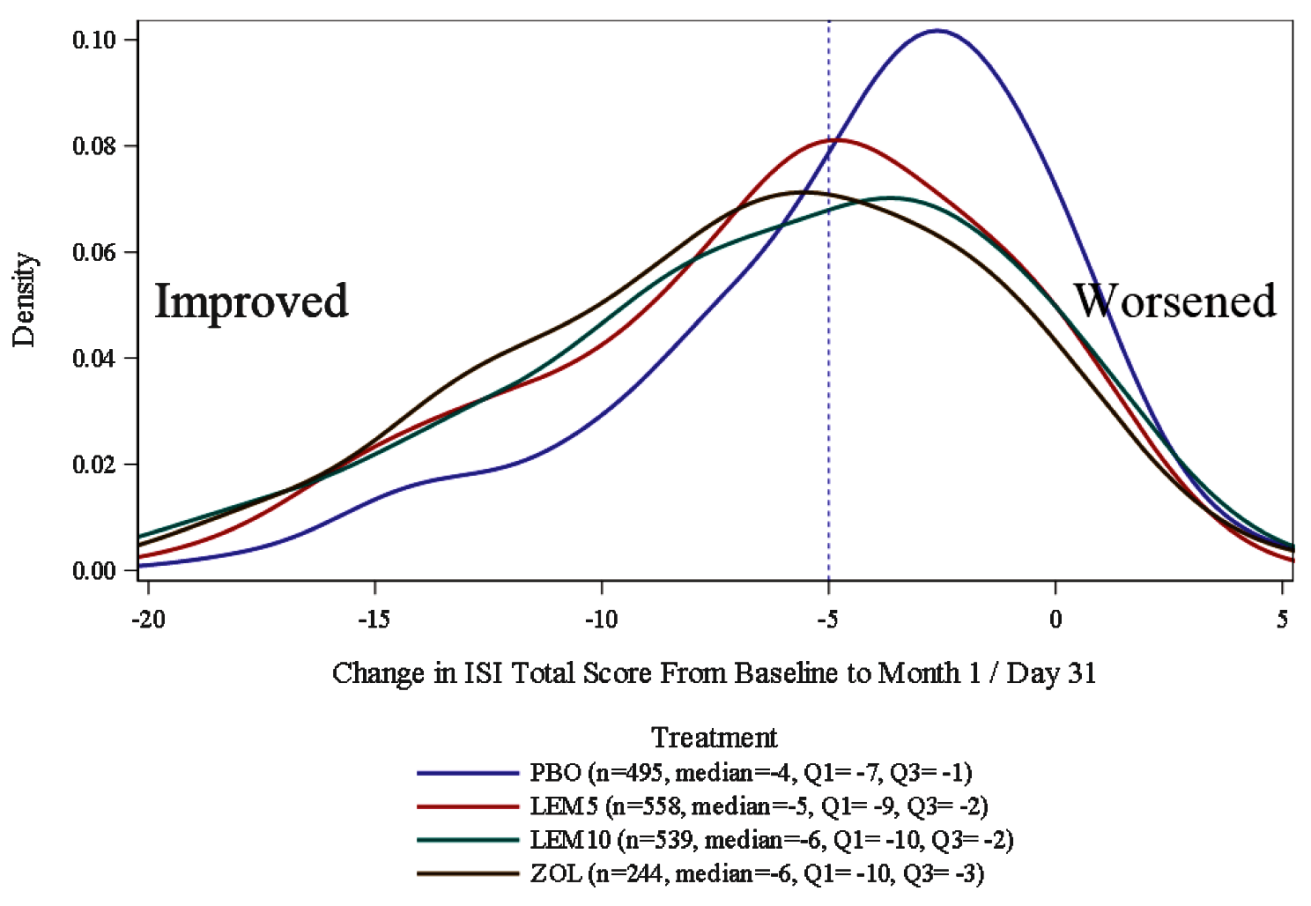
PDF plot of the change in 6-item ISI total score from baseline to Month 1/Day 31 by treatment arm. FAS (*N* = 1956). *FAS* Full Analysis Set, *ISI* Insomnia Severity Index, *LEM5* lemborexant 5 mg, *LEM10* lemborexant 10 mg, *PBO* placebo, *PDF* probability density function, *Q* quartile, *ZOL* zolpidem tartrate

The differences in change in the ISI total score between the treatment arms and PBO were examined in PDF plots to provide supporting evidence for the results above (Fig. 2). At Day 31/Month 1, the mean changes in ISI total score were −4.5 for PBO, −6.2 for LEM5, −6.3 for LEM10, and −6.6 for ZOL (Fig. 2), supporting an MWIC of −5 estimated by the triangulation approach. For example, the range of −3.6 to −4.5 represents an average change for PBO. An MWIC of −5 exceeds the average PBO response, which is appropriate, as the MWIC should not fall within the expected range of a PBO response. Using the MWIC of −5, the distribution of the responders was as follows: 119 (40.2%) for PBO, 179 (59.5%) for LEM5, and 163 (56.8%) for LEM10 for SUNRISE-2 at Month 1; and 86 (43.2%) for PBO, 156 (60.7%) for LEM5, 140 (55.6%) for LEM10 and 156 (63.9%) for ZOL for SUNRISE-1 at Day 31.

#### Summary: clinically meaningful within-individual change

**Fig. 3:**
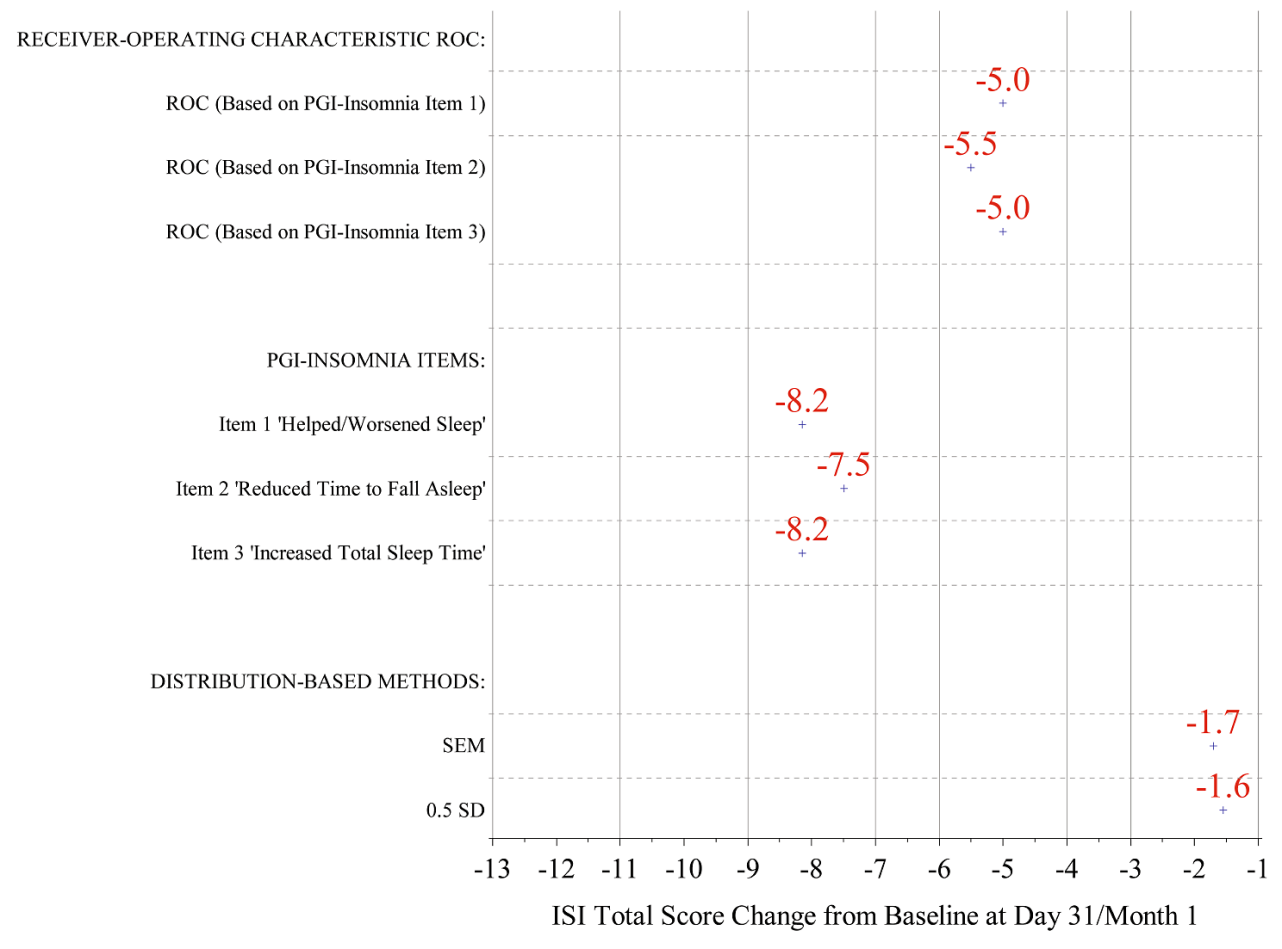
Plot of 6-item ISI total score MWIC estimates. *ISI* Insomnia Severity Index, *PGI-I* Patient Global Impression, *ROC* receiver operating characteristic, *SD* standard deviation, *SEM* standard error of measurement

The average of the MWIC estimated through triangulation was −5.0 (Fig. 3), indicating that a reduction of ≥ 5 points in total ISI score represents a clinically meaningful change in the SUNRISE-1 and SUNRISE-2 patient sample. The distribution-based method as a secondary approach demonstrated that the MWIC was on the conservative side based on this method.

## Discussion

In this study, we identified a two-factor structure without item 5 (“How noticeable to others do you think your sleep problem is in terms of impairing the quality of your life?”) as the optimal ISI structure in the context of the lemborexant clinical trial population. An MWIC of −5 was determined as the clinically meaningful change threshold for the total ISI score for this particular patient sample based on the triangulation approach.

### Model structure

The FDA guidance on PROs emphasizes the importance of having an internally consistent structure reflected in the scoring [15]. This is important because a less-than-optimal factor structure could generate additional error thus reducing the ability of a scale to detect treatment effects. We conducted this analysis to further examine the best factor structure for the ISI, as there currently is some disagreement in the literature [3–5].

We used MIs to specify structural modifications to measurement models influenced by kurtotic (or skewed) non-normal data in addition to extraction and rotation methods, as well as fit statistics commonly used in a CFA [16]. This method was used to rectify the structurally weaker 7-item solution (CFI < 0.90 and RMSEA > 0.12) associated with high MI statistics for item 5. The resulting two-factor model provided modification indices and expected parameter change (EPC) that pointed to a high residual correlation between item 5 and item 7 (MI 348.84; EPC 0.376). Removal of item 5 also removed the largest source of structural error in the resulting 6-item ISI model, which then consisted of a stronger, internally consistent daytime function scale and a symptom factor (items 1–3) that differentially characterizes the subtypes of insomnia.

We then explored effects of correlated residuals between daytime functioning and among the insomnia subtypes (identified by items 1–3). Interestingly, we found that the correlated residuals between items 5 and 7 did not affect the ISI measurement model for those with sleep-onset problems, while correlated residuals between items 5 and 6 did affect the ISI model but to a much lesser extent (MI 5.49; EPC 0.111). Once again, when item 5 was removed, the model associated with sleep-onset problems also improved. At this time, the reasons for this difference are not well understood and the findings will need further investigation.

These analyses were also repeated in the SUNRISE-1 and SUNRISE-2 trials separately (Supplementry Figs. 1 and 2). In both trials, there was a high residual correlation between item 5 and item 7, and the model fit improved with the removal of this item. Furthermore, the two-factor solution based on 6 items was supported in both trials.

In addition to the structural issues, it seemed logical to drop item 5 and keep item 7 (“To what extent do you consider your sleep problem to interfere with your daily functioning currently?”), as item 5 asks for others’ perception of the impact of insomnia on the survey responder’s quality of life, whereas item 7 asks the responder directly for their own self-perception.

The issues we noted with item 5 might explain the diversity of findings in the literature pertaining to the factor structure of the ISI.

### Clinically meaningful change threshold

In a recent FDA guidance [13], the advantages of using anchor-based methods to establish clinical meaningfulness thresholds have been reiterated. The guidance emphasizes the use of clinically relevant benchmarks and takes into account within-patient meaningful change. In this study, the PGI-I items were used as anchors to cover the multidimensional insomnia spaces and seemed to work well.

A slight drawback for the implementation of this method was too few response options. There was only one response option for the “positive” medication effect, which thus may not have included a minimal “meaningful change” but rather a range of positive responses. For example, for the PGI-I item “Helped/Worsened Sleep,” the median for the positive medication effect was −8 (interquartile range −12, −5). Thus, we additionally applied the ROC method, which is based upon modeling with the PGI-I items. In contrast to the anchor-based method, the ROC method has not been recommended by the regulators as a primary method; but it is often used to define the best performing responder threshold in terms of sensitivity and specificity. Although a distribution-based method was also used, it does not include information incorporating the patient voice and thus has not been considered a primary method for defining thresholds. As a result of a careful application of the three methods, triangulation has supported a threshold MWIC of −5 in this study. The results are not presented here for the sake of parsimony, but similar methods (anchor-based, distribution-based, and ROC methods) were used to define a meaningful threshold of -3 for each of the ISI domains.

The MWIC identified in our study using the 6-item model was different from the thresholds identified by others. Morin et al. proposed a change of −8.4 points as a moderate improvement, while Yang et al. proposed a change of −6 points as a minimally clinically important difference [6, 7]. This discrepancy might be explained by differences in the number of items included in the model and the demographics of the patient sample. First, both studies deduced the MWIC based on a 7-item ISI model, whereas we used a 6-item model. Second, the patients in our sample were on average at least 10 years older (mean age 59.3 years) than those in the Morin et al. study (mean age < 50 years) and those in the Yang et al. study (mean age 45.6 years). Furthermore, there was a higher percentage of females in our patient sample (77.6%, compared with 61.2% in the Morin et al. study).

The results of our study suggest that the factor structure, and therefore the MWIC based on this analysis, may be more robust than has been observed in the literature to date. Rather than hastily recommending that researchers switch to this version of the ISI, we suggest that further research may be valuable. This would be easy to accomplish, as the 6-item version is derived simply from dropping item 5 from the 7-item version.

## Conclusions

Our hypothesized two-factor solution had the best fit when item 5 was dropped. The final ISI model we present in this study included the following two domains: Insomnia Symptoms (items 1–3) and Daytime Functioning (items 4, 6, 7). The reduced ISI score is reliable and valid for the studied population. These results are applicable to the specific setting of the SUNRISE-1 and SUNRISE-2 clinical trial populations and demonstrate that a 6-item ISI is a viable option but is not intended to replace the 7-item ISI at this time. Further research is warranted to determine whether the factor structure we have proposed could be more sensitive to treatment effects than the legacy scale. It could also be useful to conduct additional qualitative research to see whether the rewording of selected items may improve the scale performance of the full scale and clarify the scale structure.

The best estimate of MWIC observed in the current study for the 6-item ISI total scale is −5. This estimate was derived using three common methods for establishing meaningful change thresholds. Identifying the level of change associated with a meaningful within-individual change help to stimulate further research into the clinical meaningfulness of changes associated with insomnia treatments and eventually to assess the impact of meaningful change in insomnia on the consequences of insomnia, such as cognitive deficits, emotional distress, and mood disorders.

### Electronic supplementary material

Below is the link to the electronic supplementary material.

Supplementary Material 1

## Data Availability

The data that support the findings of this study are available from the corresponding author upon reasonable request.

## Abbreviations

CFA: Confirmatory factor analysis
CFI: Comparative fit index
CI: Confidence interval
EPC: Expected parameter change
FAS: Full analysis set
FDA: Food and Drug Administration
ISI: Insomnia Severity Index
LEM: Lemborexant
MWIC: Meaningful within-individual change
MI: Modification index
PBO: Placebo
PDF: Probability density function
PGI-I: Patient Global Impression—Insomnia
PRO: Patient-reported outcome
RMSEA: Root mean square error of approximation
ROC: Receiver operating characteristic
SD: Standard deviation
SEM: Standard error of measurement
TST: Total sleep time
WRMR: Weighted root mean square residual
ZOL: Zolpidem tartrate
