# Supplementary material for: Re-examining the factor structure of the Insomnia Severity Index (ISI) and defining the meaningful within-individual change (MWIC) for subjects with insomnia disorder in two phase III clinical trials of the efficacy of lemborexant
Source: J Patient Rep Outcomes. 2024 Jun 29;8:65. doi: 10.1186/s41687-024-00744-6 (PMC11217251; doi:10.1186/s41687-024-00744-6)
Supplement: Supplementary file 1 — Supplementary Material 1 [file 41687_2024_744_MOESM1_ESM.docx]

**Supplemental Table 1** Description of baseline ISI items, FAS (N=1956)

|  | SUNRISE-1 (N=1006) | SUNRISE-2 (N=950) | Pooled trials (N=1956) |
| --- | --- | --- | --- |
| **Factor 1** | | | |
| 1. Difficulty falling asleep |  |  |  |
| None | 29 (2.9%) | 26 (2.7%) | 55 (2.8%) |
| Mild | 79 (7.9%) | 48 (5.1%) | 127 (6.5%) |
| Moderate | 368 (36.6%) | 264 (27.8%) | 632 (32.3%) |
| Severe | 423 (42.0%) | 466 (49.1%) | 889 (45.4%) |
| Very severe | 107 (10.6%) | 146 (15.4%) | 253 (12.9%) |
| Missing | 0 (0.0%) | 0 (0.0%) | 0 (0.0%) |
| 2. Difficulty staying asleep |  |  |  |
| None | 1 (0.1%) | 5 (0.5%) | 6 (0.3%) |
| Mild | 10 (1.0%) | 23 (2.4%) | 33 (1.7%) |
| Moderate | 223 (22.2%) | 222 (23.4%) | 445 (22.8%) |
| Severe | 580 (57.7%) | 526 (55.4%) | 1106 (56.5%) |
| Very severe | 192 (19.1%) | 174 (18.3%) | 366 (18.7%) |
| Missing | 0 (0.0%) | 0 (0.0%) | 0 (0.0%) |
| 3. Problems waking up too early |  |  |  |
| None | 19 (1.9%) | 37 (3.9%) | 56 (2.9%) |
| Mild | 74 (7.4%) | 100 (10.5%) | 174 (8.9%) |
| Moderate | 353 (35.1%) | 301 (31.7%) | 654 (33.4%) |
| Severe | 447 (44.4%) | 378 (39.8%) | 825 (42.2%) |
| Very severe | 113 (11.2%) | 134 (14.1%) | 247 (12.6%) |
| Missing | 0 (0.0%) | 0 (0.0%) | 0 (0.0%) |
| **Factor 2** | | | |
| 4. How satisfied/dissatisfied are you with your current sleep pattern? |  |  |  |
| Very satisfied | 3 (0.3%) | 0 (0.0%) | 3 (0.2%) |
| Satisfied | 10 (1.0%) | 4 (0.4%) | 14 (0.7%) |
| Moderately satisfied | 48 (4.8%) | 18 (1.9%) | 66 (3.4%) |
| Dissatisfied | 553 (55.0%) | 529 (55.7%) | 1082 (55.3%) |
| Very dissatisfied | 392 (39.0%) | 399 (42.0%) | 791 (40.4%) |
| Missing | 0 (0.0%) | 0 (0.0%) | 0 (0.0%) |
| 5. How noticeable to others do you think your sleep problem is in term of impairing the quality of your life? |  |  |  |
| Not at all noticeable | 109 (10.8%) | 36 (3.8%) | 145 (7.4%) |
| A little | 224 (22.3%) | 138 (14.5%) | 362 (18.5%) |
| Somewhat | 377 (37.5%) | 417 (43.9%) | 794 (40.6%) |
| Much | 233 (23.2%) | 302 (31.8%) | 535 (27.4%) |
| Very much noticeable | 63 (6.3%) | 57 (6.0%) | 120 (6.1%) |
| Missing | 0 (0.0%) | 0 (0.0%) | 0 (0.0%) |
| 6. How worried/distressed are you about your current sleep problem? |  |  |  |
| Not at all worried | 16 (1.6%) | 1 (0.1%) | 17 (0.9%) |
| A little | 63 (6.3%) | 24 (2.5%) | 87 (4.4%) |
| Somewhat | 264 (26.2%) | 232 (24.4%) | 496 (25.4%) |
| Much | 422 (41.9%) | 488 (51.4%) | 910 (46.5%) |
| Very much worried | 241 (24.0%) | 205 (21.6%) | 446 (22.8%) |
| Missing | 0 (0.0%) | 0 (0.0%) | 0 (0.0%) |
| 7. To what extent do you consider your sleep problem to interfere with your daily functioning (e.g. daytime fatigue, mood, ability to function at work/daily chores, concentration, memory, mood, etc.) currently? |  |  |  |
| Not at all interfering | 35 (3.5%) | 8 (0.8%) | 43 (2.2%) |
| A little | 128 (12.7%) | 39 (4.1%) | 167 (8.5%) |
| Somewhat | 390 (38.8%) | 362 (38.1%) | 752 (38.4%) |
| Much | 346 (34.4%) | 438 (46.1%) | 784 (40.1%) |
| Very much interfering | 107 (10.6%) | 103 (10.8%) | 210 (10.7%) |
| Missing | 0 (0.0%) | 0 (0.0%) | 0 (0.0%) |

FAS, full analysis set; ISI, Insomnia Severity Index

**a.**


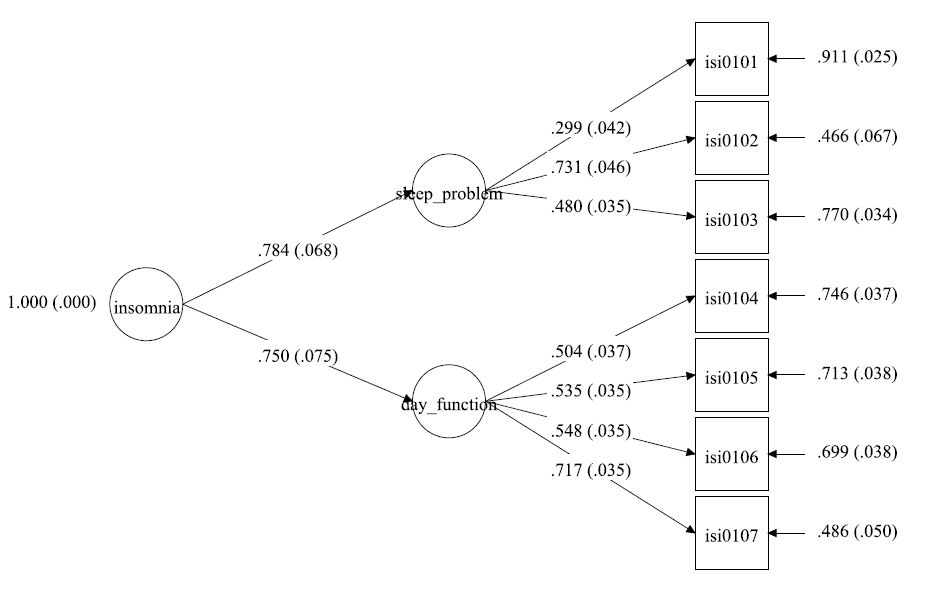


**b.**


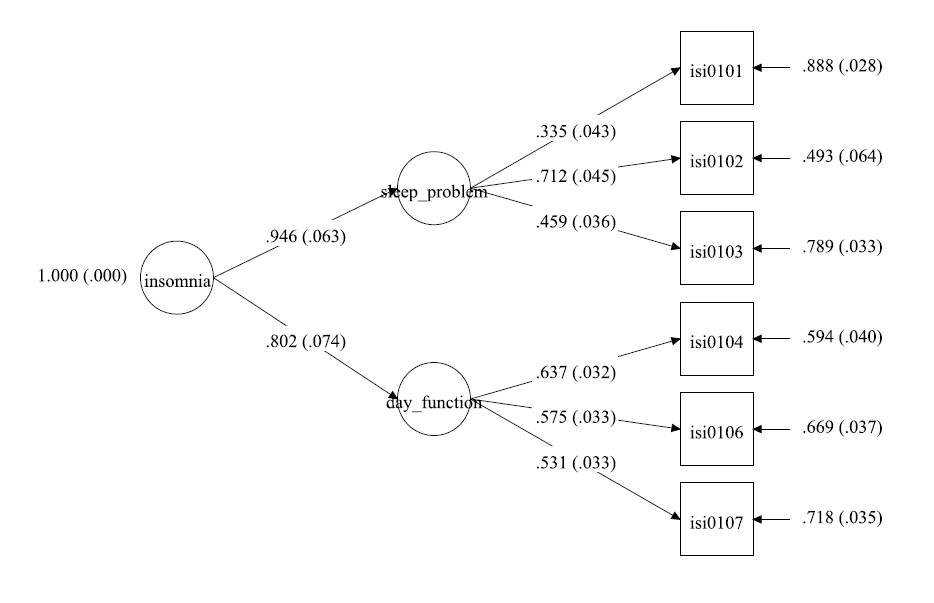


**Supplemental Figure 1.** Two-factor confirmatory factor analysis models from SUNRISE-1 (N=1006) **a** with 7 items; **b** with item 5 removed. The figure shows the factor loadings on each item of the ISI.

7-item fit statistics: CFI = 0.800; RMSEA (90% CI) = 0.129 (0.115 – 0.145); MI for items 5 and 7 = 140.33

6-item fit statistics: CFI= 0.913; RMSEA (90% CI) = 0.092 (0.073 – 0.112)


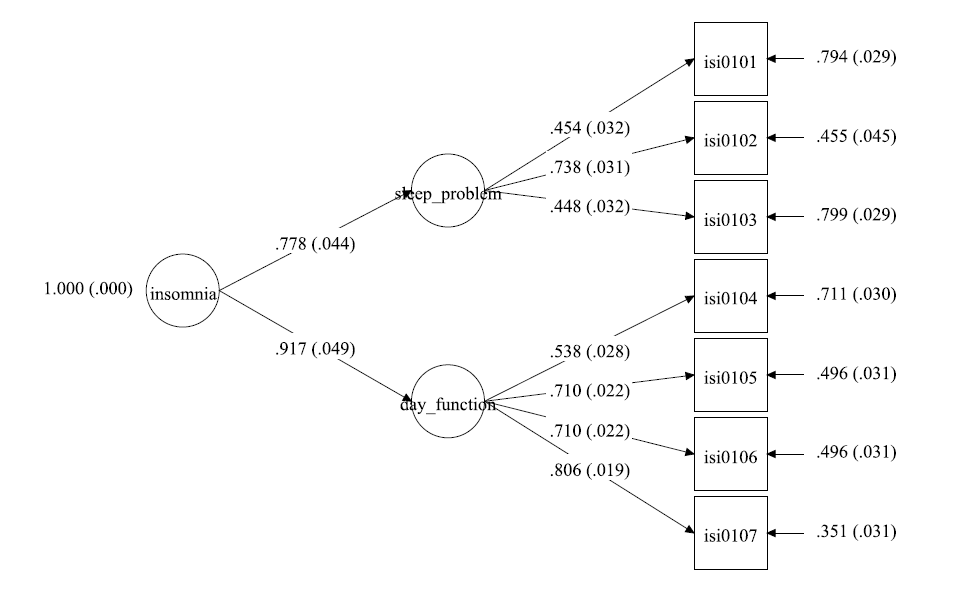
**a.**

**b.**


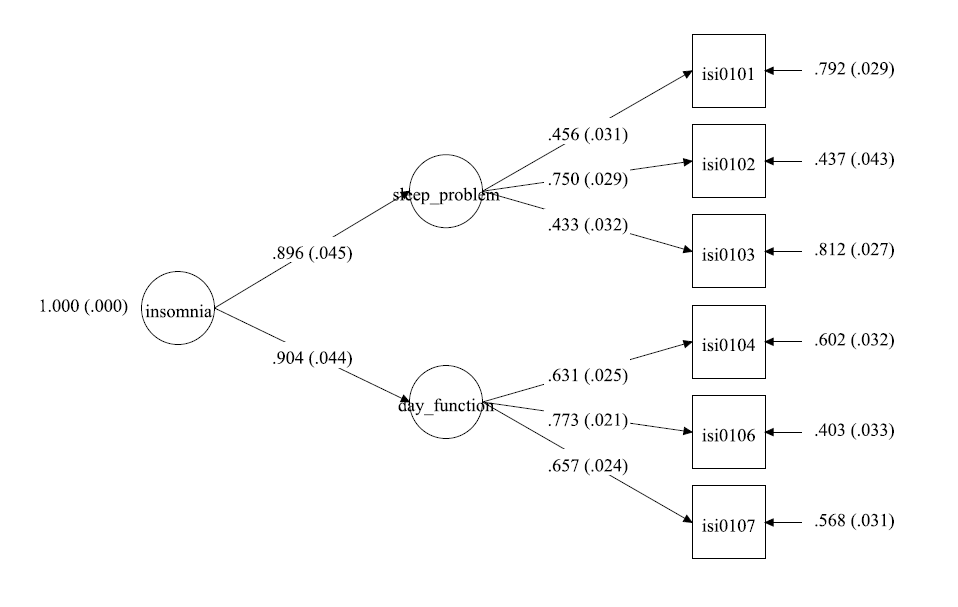


**Supplemental Figure 2**. Two-factor confirmatory factor analysis models from SUNRISE-2 (N=950) **a** with 7 items; **b** with item 5 removed. The figure shows the factor loadings on each item of the ISI.

7-item fit statistics: CFI = 0.895; RMSEA (90% CI) = 0.122 (0.108 – 0.137); MI for items 5 and 7 = 204.92

6-item fit statistics: CFI= 0.982; RMSEA (90% CI) = 0.054 (0.035 – 0.074)
